# Moderate alcohol-associated hepatitis: A real-world multicenter study

**DOI:** 10.1097/HC9.0000000000000673

**Published:** 2025-03-24

**Authors:** Francisco Idalsoaga, Luis Antonio Díaz, Winston Dunn, Heer Mehta, Karen Muñoz, Vicente Caldentey, Jorge Arnold, Gustavo Ayares, Rokhsana Mortuza, Shiv K. Sarin, Rakhi Maiwall, Wei Zhang, Steve Qian, Douglas Simonetto, Ashwani K. Singal, Mohamed A. Elfeki, Carolina Ramirez-Cadiz, Gurpreet Malhi, Adan Ahmed, Hoomam Homsi, Zinia Abid, Joaquín Cabezas, Victor Echavarría, Maria Poca, German Soriano, Berta Cuyas, Meritxell Ventura Cots, María Fátima Higuera-De La Tijera, Maria Ayala-Valverde, Diego Perez, Jaime Gomez, Juan G Abraldes, Mustafa Al-Karaghouli, Prasun K. Jalal, Mohamad A. Ibrahim, Guadalupe García-Tsao, Daniela Goyes, Lubomir Skladaný, Daniel J. Havaj, Karolina Sulejova, Svetlana Adamcova Selcanova, Diego Rincón, Kristina R. Chacko, Juan C. Restrepo, Pamela Yaquich, Luis G. Toro, Vijay Shah, Marco Arrese, Patrick S. Kamath, Ramon Bataller, Juan Pablo Arab

**Affiliations:** 1 Departamento De Gastroenterología, Escuela De Medicina, Pontificia Universidad Católica De Chile, Santiago, Chile; 2 Division of Gastroenterology and Hepatology, Department of Medicine, Western University & London Health Sciences Centre, London, Canada; 3 Division of Gastroenterology and Hepatology, Department of Medicine, MASLD Research Center, University of California San Diego, San Diego, California, USA; 4 Division of Gastroenterology, Department of Medicine, University of Kansas Medical Center, Kansas City, Missouri, USA; 5 Escuela de Medicina, Pontificia Universidad Católica de Chile, Santiago, Chile; 6 Department of Hepatology, Institute of Liver and Biliary Sciences, New Delhi, India; 7 Department of Medicine, Gastroenterology Unit, Massachusetts General Hospital, Harvard Medical School, Boston, Massachusetts, USA; 8 Division of Gastroenterology and Hepatology, Department of Medicine, University of Florida, Gainesville, Florida, USA; 9 Division of Gastroenterology and Hepatology, Department of Medicine, Mayo Clinic, Rochester, Minnesota, USA; 10 Division of Gastroenterology, Department of Medicine, Hepatology and Nutrition, University of Louisville School of Medicine, Louisville, Kentucky, USA; 11 Department of Anesthesiology, Virginia Commonwealth University School of Medicine, Richmond, Virginia, USA; 12 Gastroenterology and Hepatology Department, University Hospital Marqués de Valdecilla, Santander, Spain; 13 Research Institute Valdecilla (Idival), Santander, Spain; 14 Department of Gastroenterology, Hospital de la Santa Creu i Sant Pau, Institut de Recerca Hospital de Sant Pau-IIB Sant Pau, Universitat Autònoma de Barcelona, CIBERehd, Barcelona, Spain; 15 Department of Hepatology, Liver Unit, Hospital Vall D’hebron, Universitat Autonoma Barcelona, Ciberehd, Barcelona, Spain; 16 Servicio de Gastroenterología, Hospital General De México, Universidad Nacional Autónoma de México, México; 17 Servicio Medicina Interna, Hospital El Pino, Santiago, Chile; 18 Division of Gastroenterology, Department of Medicine, Liver Unit, University of Alberta, Edmonton, Alberta, Canada; 19 Department of Gastroenterology and Hepatology, Baylor College of Medicine, Houston, Texas, USA; 20 Section of Digestive Diseases, Yale University School of Medicine/VA-CTt Healthcare System, New Haven/West Haven, Connecticut, USA; 21 Division of Hepatology, Gastroenterology and Liver Transplantation, Department of Internal Medicine II, Slovak Medical University, F. D. Roosevelt University Hospital, Banska Bystrica, Slovak Republic; 22 Liver Unit, Department of Digestive Diseases Hospital General Universitario Gregorio Marañón Madrid, Spain; 23 Ciberehd Centro De Investigación Biomédica En Red De Enfermedades Hepáticas Y Digestivas Madrid, Spain; 24 Division of Gastroenterology and Hepatology, Department of Medicine, Montefiore Medical Center, Bronx, New York, USA; 25 Department of Internal Medicine, Unidad de Hepatología del Hospital Pablo Tobon Uribe, Grupo de Gastrohepatología de la Universidad de Antioquia, Medellín, Colombia; 26 Departamento de Gastroenterología, Hospital San Juan De Dios, Santiago, Chile; 27 Hepatology and Liver Transplant Unit, Hospitales de San Vicente Fundación de Medellín y Rionegro, Colombia; 28 Liver Unit, Hospital Clinic, Barcelona, Spain; 29 Institut d’Investigacions Biomèdiques August Pi i Sunyer (IDIBAPS), Barcelona, Spain; 30 Division of Gastroenterology, Hepatology, and Nutrition, Department of Internal Medicine, Virginia Commonwealth University School of Medicine, Richmond, Virginia, USA

**Keywords:** alcohol, alcohol-associated hepatitis, alcohol-associated liver disease, cirrhosis, end-stage liver disease, outcome prediction

## Abstract

**Background:**

Severe alcohol-associated hepatitis (sAH) is a well-characterized disease with high short-term mortality. However, there is limited research on those with a “less severe condition” (moderate AH). This study aims to characterize in-depth patients with moderate AH (mAH), including the performance of mortality scoring systems, key prognostic factors, and survival over time.

**Methods:**

A multicenter retrospective cohort study (2009–2019) included patients with mAH (MELD score ≤20 at admission). Cox regression and receiver operating characteristic curves with AUC were used for analysis.

**Results:**

We included 1845 patients with AH (20 centers, 8 countries) between 2009 and 2019. mAH was defined as a MELD score ≤20 at admission. Twenty-four percent met the criteria for an mAH episode. Patients with mAH tend to be older and have a higher proportion of females, with a median MELD of 17 (15–19), Maddrey discriminant function (mDF) of 33 (22–40), the trajectory of serum bilirubin of 0.83 (0.60–1.21), and neutrophil-to-lymphocyte ratio (NLR) of 5 (2.96–8.60). The primary causes of death in mAH included multiple organ failure (34.1%) and infections (16.6%). The cumulative survival rates at 30, 90, and 180 days were 94.3%, 90.4%, and 88.2%, respectively. In multivariable analysis, age was the only significant predictor of 30-day mortality (HR 1.49, 95% CI: 1.27–1.76, *p*<0.001). Mortality prediction models showed poor performance, with AUC for MELD (0.671), mDF (0.726), trajectory of serum bilirubin (0.733), and NLR (0.697).

**Conclusions:**

Patients with moderate AH exhibited a mortality of 11.8% at 6 months, primarily driven by multiple organ failure and infections. These patients also exhibit a different clinical profile compared to those with sAH. Tailored models and therapeutic strategies are needed to improve long-term outcomes in mAH.

## Graphical abstract

**Figure:**
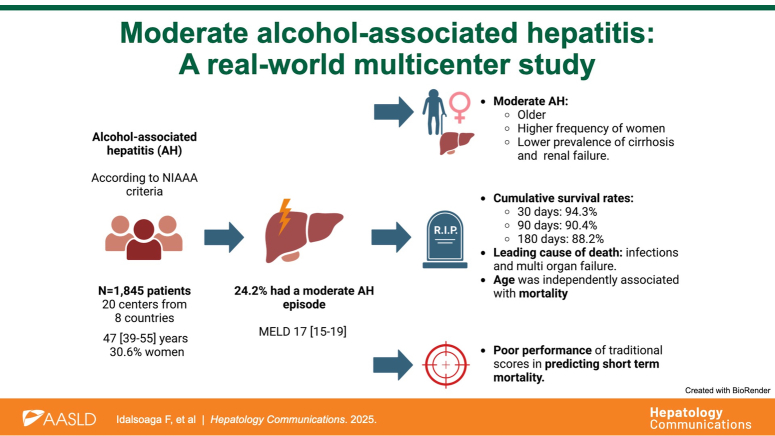

## INTRODUCTION

Alcohol-associated liver disease (ALD) is a growing global health problem.^1^ The high morbidity and mortality, as well as the associated health care burden, accelerated during the COVID-19 pandemic, becoming an even more concerning factor among young individuals and women.^2^ Differences across races have also been observed, with a greater impact on Native Americans and Hispanics, who are experiencing some of the largest increases in ALD rates.^3^ Additionally, regions such as Europe and the Americas, have the highest alcohol-attributable deaths and disability-adjusted life-years attributable to alcohol intake worldwide.^4^ This is particularly worrisome in areas with important social inequities and other impaired determinants of health.^5^^–^^7^ Severe alcohol-associated hepatitis (sAH) represents an acute presentation of ALD and is a fatal condition with high short-term mortality.^8^ This condition is characterized by a sudden onset of jaundice, malaise, decompensated liver disease, and coagulopathy.^9^ Individuals with sAH usually develop bacterial infections, acute-on-chronic liver failure, and multiorgan failure.^10^ Consequently, patients with sAH could have a mortality of 30% at 28 days, along with long-term mortality that could be up to 50% at one year.^11^^–^^13^

In this context, AH is classified as Severe AH or moderate AH (nonsevere AH). The severity of AH is commonly assessed using the Maddrey discriminant function (mDF)^14^ or the Model for End-Stage Liver Disease (﻿MELD) score﻿^15^ in clinical practice. Severe AH (sAH) is defined by an mDF of ≥32 or a MELD score >20, while nonsevere or moderate AH (mAH) is characterized by mDF <32 or a MELD score ≤20 (1).

In sAH, MELD has a better performance than mDF in classifying patients at a higher risk of short-term mortality.^15^^,^^16^ While the incidence of mAH remains somewhat elusive, it appears to surpass that of its severe counterpart. Despite the predominant focus on sAH in numerous investigations, recent studies have shown that an episode of mAH could also reach mortality rates of at least 6% and 12% at 28 days and 90 days, respectively.^17^^,^^18^ The natural history, prognostic factors, and management of mAH are not well established, given that the vast majority of studies so far have focused on sAH. The approach to its treatment rests upon 3 pivotal cornerstones: sustained abstinence from alcohol over an extended period, provision of essential nutritional support, and adept handling of the complications stemming from liver impairment.^13^

Unfortunately, mAH remains inadequately understood concerning its natural progression and the most effective management and treatment strategies, despite its substantial morbidity and mortality.^8^ Only a limited number of studies have investigated the natural course of this disease during the past few decades, leaving a significant knowledge gap regarding risk stratification and therapeutic approaches in this scenario. In a recent retrospective study comparing patients with mAH and sAH, patients with mAH were older and had a lower prevalence of cirrhosis compared to those with sAH. It was also found that age, acute kidney injury, and corticosteroid use were associated with an increased risk of 90-day mortality.^18^

Another area with limited evidence is the role of different scoring systems in classifying the severity of this condition. Various scores, such as MELD, mDF, trajectory of serum bilirubin (TSB), and the neutrophil-to-lymphocyte ratio (NLR), have been validated for sAH.^14^^,^^15^^,^^19^^,^^20^ However, their validity and utility in mAH remain uncertain. Consequently, this study aims to characterize patients with mAHs on a global scale, assess the performance of traditional scoring systems to predict mortality, identify relevant prognostic factors, and evaluate short to medium-term survival outcomes.

## METHODS

### Study design and participants

We conducted a retrospective cohort analysis using registry data, including individuals admitted for moderate and severe AH based on the well-defined clinical standards outlined by the National Institute on Alcohol Abuse and Alcoholism (NIAAA).^21^ mAH was defined by a MELD score of <21, given the evidence of its superior performance compared to the mDF﻿.^15^^,^^16^ Patients included in the study had a history of alcohol consumption (>60 g/d for men and >40 g/d for women), aspartate aminotransferase levels <400 U/L with an aspartate aminotransferase/alanine aminotransferase ratio >1.5, serum GGT >80 mg/dL, serum total bilirubin >3.0 mg/dL, and abnormal coagulation tests (prolonged prothrombin time and/or international normalized ratio [INR] values). The diagnosis of cirrhosis was determined based on medical history and imaging methods, including ultrasound, transient elastography, computed tomography, and magnetic resonance imaging.

Exclusion criteria included individuals under 18 years old, pregnant individuals, those with aspartate aminotransferase and/or alanine aminotransferase levels exceeding 400 IU/mL, patients who had abstained from alcohol for more than 60 days before presentation, cases of DILI, ischemic hepatitis, biliary duct blockage, viral hepatitis, autoimmune hepatitis, or Wilson disease. Additionally, we excluded cases of hepatocellular carcinoma beyond the Milan criteria, other neoplasms with a life expectancy of <6 months, or a history of severe extrahepatic conditions indicating a survival chance of less than 6 months.

### Data collection

We conducted a retrospective analysis by reviewing the medical records of hospitalized patients diagnosed with severe AH using the previously outlined criteria, covering the period from January 2009 to January 2019. We documented laboratory results conducted during admission, including MELD, mDF, trajectory of serum bilirubin (TSB), NLR, infections, mortality, and causes of death at 90 days. In the TSB, patients categorized as “fast fallers” (bilirubin <0.8 x admission value at day 7) were deemed not to benefit from corticosteroid treatment or were nonsevere patients, as per the original publication of this score.^19^ Regarding NLR, values between 5 and 8 were considered indicative of responsive patients.^20^ It was not possible to obtain a record of the amount of alcohol consumption, whether they achieved abstinence after episodes of AH, or if they needed to start therapy for alcohol use disorder. Access to this data was restricted to the research team, and we obtained a waiver of informed consent from each participating center. The project was approved by the ethics committee of the Pontificia Universidad Católica de Chile. This study was conducted under Good Clinical Practice guidelines, and the Declaration of Helsinki and Istanbul.

### Statistical analysis

We assessed the distribution of data using the Shapiro-Wilk test. Continuous data were characterized by presenting the mean and SD, while non-normally distributed data using the median and IQR. Nominal data were depicted using percentages. Comparison of numerical variables with normal distribution employed the Chi-squared test and either Student *t* test or ANOVA. Nonparametric tests were applied for numerical variables that did not follow a normal distribution. In instances involving multiple proportions, the *p* value was determined using binomial regression with a log link function.

The primary endpoint of this study was 30-day mortality among patients with mAH. Secondary endpoints included 90-day and 180-day mortality, key variables associated with increased mortality in mAH, and the evaluation of the performance of traditional models in predicting short-term mortality. The use of competing risk models was evaluated, treating transplantation as a censoring event. However, no differences were observed in the cumulative incidence, so it was decided not to use this type of model (Supplemental Figure S1, http://links.lww.com/HC9/B939). To analyze our time-to-morality outcome, we used a Cox proportional hazards regression available in the survival package available for R adjusting by age, sex, MELD score, mDF score, plasma creatinine, total bilirubin, sodium, albumin, international normalized ratio, use of corticoids, history of cirrhosis, and infections. We scaled all continuous variables to have a mean of 0 and an SD of 1. The log HRs are then interpreted as the change in the log hazard associated with a 1 SD change in the variable. We used complete case analysis and removed those individuals with missing data from the analysis. To evaluate if there were variations in survival among patients with mAH with different MELD scores, patients were stratified with MELD scores of 8, 12, 15, 18, and 20. This approach aimed to determine individuals with significantly lower mortality—as a “mild” AH episode. Receiver operating characteristic curves were used to evaluate the performance of different scores in predicting mortality in patients with moderate AH, and the AUC was calculated for each score at different follow-up times. Statistical analysis was performed with R software version 4.3.1. A *p* value of <0.05 was considered statistically significant.

## RESULTS

### Baseline characteristics of the cohort

We included 1845 patients with AH from 20 centers across 8 countries spanning 3 continents (Supplemental Table S1, http://links.lww.com/HC9/B939). The median age was 47 years [IQR: 39–55], with 565 (30.6%) patients being women. Among them, 959 (53%) patients were Caucasian, 442 (24.4%) were Indian, 122 (6.7%) were Hispanic-Latino, and 115 (6.3%) were Black. A total of 1040 (74.8%) of the patients had a prior history of cirrhosis, and 90 (4.8%) underwent liver transplantation. The median MELD score on admission was 24 (20–31), and the mDF was 57.6 (40–83). About 796 (43.2%) of the patients received corticosteroids, and the Lille score at 4 and 7 days were 0.44 (0.16–0.77) and 0.42 (0.12–0.79), respectively. The median TSB score was 0.91 (0.70–1.17), and the NLR was 6.82 (3.85–11.19) (Table 1). Of the total cohort, 46.2% (403) presented with an infection at admission; however, there was a significant percentage (52%) of missing data.

**TABLE 1:** Baseline characteristics of patients

	Global (N=1845)	Severe AH (N=1399)	Moderate AH (N=446)	*p*
Age (mean)	47 [39–55]	47 [38–55]	49 [40–56]	0.008
Sex (women), N (%)	565 (30.6)	406 (29)	159 (35.6)	0.008
Race/ethnicity﻿, N (%)				
White or Caucasian	978 (53)	707 (50.5)	273 (61.3)	<0.001
Indian	450 (24.4)	416 (29.7)	29 (6.7)	
Hispanic or Latino	123 (6.7)	68 (4.8)	57 (12.9)	
Black	116 (6.3)	83 (6)	34 (7.4)	
Mestizo	65 (3.5)	49 (3.5)	15 (3.3)	
American Indian	28 (1.5)	16 (1.2)	12 (2.6)	
Asian Pacific Islander	20 (1.1)	14 (1)	7 (1.4)	
Other	65 (3.5)	46 (3.3)	19 (4.4)	
MELD	24 [20–31]	27 [23–34]	17 [15–19]	<0.001
Maddrey discriminant function	57.6 [40–83]	68 [51–91]	33 [22–40]	<0.001
Lille-4 Score	0.44 [0.16–0.77]	0.53 [0.22–0.82]	0.15 [0.05–0.38]	<0.001
Lille-7 Score	0.42 [0.13–0.79]	0.52 [0.19–0.85]	0.16 [0.05–0.36]	<0.001
Trajectory of serum bilirubin	0.91 [0.70–1.17]	0.98 [0.71–1.17]	0.83 [0.60–1.21]	0.003
Neutrophil-to-leukocyte ratio	6.82 [3.85–11.19]	7.5 [4.11–12]	5 [2.96–8.6]	<0.001
Cirrhosis, N (%)	1.040 (74.8)	770 (76.3)	270 (71)	0.044
Use corticoids, N (%)	796 (43.2)	666 (47.6)	130 (29.1)	<0.001
AKI at admission	550 (29.8)	532 (38)	18 (4)	<0.001
Liver transplant rate, N (%)	90 (4.8)	78 (5.5)	12 (2.6)	0.057
Laboratory testing at admission				
Total bilirubin (mg/dL)	13.4 [7.1–23.7]	17.6 [10.7–26.6]	5.3 [3.6–7.8]	<0.001
International normalized ratio (INR)	1.8 [1.5–2.3]	2.0 [1.7–2.45]	1.5 [1.2–1.7]	<0.001
Creatinine (mg/dL)	0.84 [0.59–1.4]	1 0.61–1.89]	0.65 [0.51–0.84]	<0.001
Sodium (mEq/L)	132 [128–136]	132 [127–135]	134 [130–137]	<0.001
Albumin (g/dL)	2.6 [2–3]	2.4 [2–3]	2.9 [2.4–3.3]	<0.001

Abbreviations: AKI, acute kidney injury; INR, international normalized ratio; MELD, Model for End-Stage Liver Disease.

A total of 446 (24.17%) patients met the criteria for mAH. Among these patients, the median age was 49 years (40–56), with 159 (35.6%) being women, and 255 (61.3%) were Caucasian. The MELD score was 17 (15–19), and the mDF was 33 (22–40). The median TSB and NLR scores were 0.83 (0.60–1.21) and 5 (2.96–8.60), respectively. In patients with mAH compared to those with sAH, individuals with mAH were older (49 y [40–56] vs. 47 y [38–55], *p*=0.008), and a higher percentage were women (35.6% vs. 29%; *p*=0.008). Moreover, the prevalence of cirrhosis was lower in patients with mAH (71% vs. 76.3%, *p*=0.044), as well as the incidence of renal failure upon admission (4% vs. 38%, *p*<0.001). The use of corticosteroids was also less frequent in patients with mAH (29.1% vs. 47.6%, *p*<0.001). The percentage of liver transplantation at the end of follow-up (18 mo) tended to be lower in the mAH group (2.6% vs. 5.5%, *p*=0.057), although this difference did not reach statistical significance. In addition, patients with mAH had lower scores for MELD (*p*<0.001), mDF (*p*<0.001), Lille-4 (*p*<0.001), Lille-7 (*p*<0.001), TSB (*p*<0.001), and NLR (*p*<0.003) compared to patients with sAH (Table 1).

### Survival and variables associated with mortality

**FIGURE 1:**
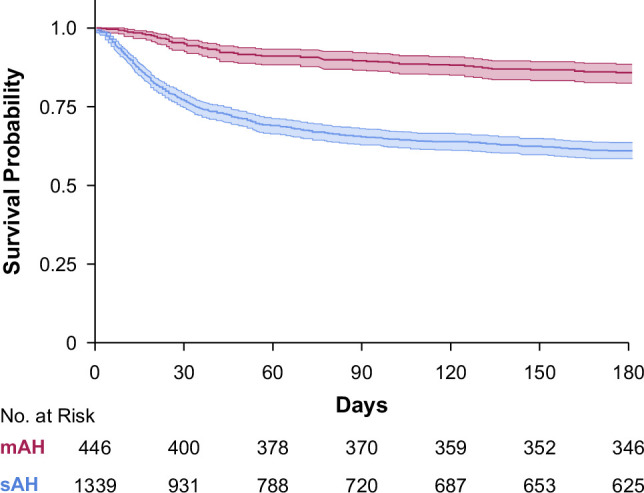
Kaplan-Meier curve of survival in patients with moderate and severe alcohol-associated hepatitis. Abbreviations: mAH, moderate alcohol-associated hepatitis, sAH, severe alcohol-associated hepatitis.

In mAH, the cumulative survival rates at 30, 90, and 180 days were 94.3% (95% CI: 91.9–96.8), 90.4% (CI: 87.3–93.6), and 88.2% (95% CI: 84.8–91.8), respectively (Figure 1). In the whole cohort, the causes of death were 149 (34.1%) due to multiple organ failure, 73 (16.6%) infections, 44 (10%) esophageal variceal bleeding, and 18 (4.1%) acute renal failure. In severe AH, although multiorgan failure (36.2% vs. 24%), infections (17.4% vs. 13.3%), and esophageal varices bleeding (10.5% vs. 8%) tend to be more frequent compared to mAH, no significant differences were found (*p*=0.053).

**FIGURE 2:**
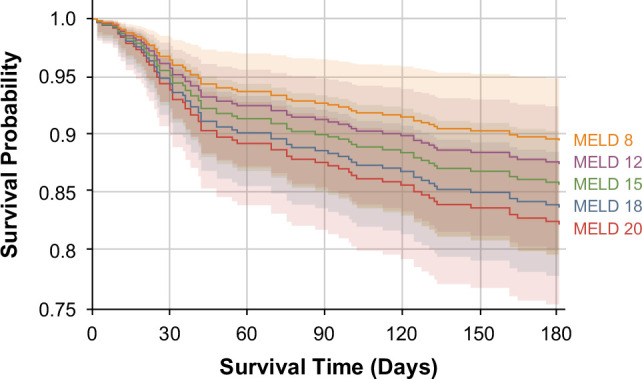
Adjusted survival curves stratified by MELD. The model is adjusted for age, male gender, history of cirrhosis, albumin at admission, plasma sodium at admission, and use of corticosteroids.

The univariate analysis identified age (HR 1.47, 95% CI: 1.26–1.70, *p*<0.001) and MELD score (HR 1.24, 95% CI: 1.06–1.46, *p*=0.006) as significant predictors of 30-day mortality. However, in the multivariate analysis, age remained a significant predictor (HR 1.49, 95% CI: 1.27–1.76, *p*<0.001), whereas MELD score did not (HR 1.41, 95% CI: 0.94–1.37, *p*=0.160). Other variables, including sex, cirrhosis, corticosteroid use, albumin levels, and sodium at admission, did not show significant associations with mortality in either analysis (Table 2). When stratifying survival by MELD score, 30-, 90-, and 180-day survival rates were 94.4%, 90.5%, and 84.9% for MELD 8; 95.7%, 91.3%, and 87.4% for MELD 12 95.1%, 90%, and 85.6% for MELD 15; 94.4%, 88.6%, and 83.6% for MELD 18; and 93.9%, 87.6%, and 82.2% for MELD 20, respectively (Figure 2 and Supplemental Table S2, http://links.lww.com/HC9/B939).

**TABLE 2:** Multivariable Cox-regression model for 30-day mortality

	Univariate analysis		Multivariate analysis	
Variables	HR (95% CI)	*p*	HR (95% CI)	*p*
Age (y)	1.47 (1.26–1.70)	<0.001	1.49 (1.27–1.76)	<0.001
Sex (male)	1.01 (0.74–1.37)	0.944	1.21 (0.81–1.54)	0.485
MELD	1.24 (1.06–1.46)	0.006	1.41 (0.94–1.37)	0.160
Cirrhosis	1.32 (0.92–1.88)	0.121	0.98 (0.67–1.64)	0.969
Corticosteroids use	1.14 (0.83–1.57)	0.392	0.87 (0.62–1.23)	0.449
Albumin at admission	0.86 (0.74–1.00)	0.063	0.86 (0.72–1.03)	0.106
Sodium at admission	1.04 (0.88–1.22)	0.606	1.08 (0.91–1.28)	0.345

### Performance of scoring systems predicting mAH mortality

In the assessment of the performance of various scores for predicting 30-day mortality, the scores had similar AUC values: MELD at 0.671, mDF at 0.726, TSB at 0.733, and NLR at 0.697 (Supplemental Figure S2, http://links.lww.com/HC9/B939). When evaluating performance at 90 days, the AUC values were 0.700 for MELD, 0.725 for mDF, 0.726 for TSB, and 0.710 for NLR. For predicting 180-day mortality, the AUC values were 0.703 for MELD, 0.711 for mDF, 0.723 for TSB, and 0.706 for NLR (Table 3). No significant differences were observed when comparing the different scores with MELD at 30, 90, and 180 days (mDF at 30 d *p*=0.105, 90 d *p*=0.244, 180 d *p*=0.493/TSB at 30 d *p*=0.074, 90 d *p*=0.300, 180 d *p*=0.149/NLR at 30 d *p*=0.405, 90 d *p*=0.662, 180 d *p*=0.810).

**TABLE 3:** Values of AUC when evaluating the performance of different scores to predict 30-day mortality

	Score			
Days	MELD (95% IC)	mDF	TSB	NLR
**30** **d**	0.671 (0.56–0.75)	0.726 (0.62–0.81)	0.733 (0.63–0.80)	0.697 (0.60–0.79)
**90** **d**	0.700 (0.63–0.76)	0.725 (0.64–0.79)	0.726 (0.65–0.79)	0.710 (0.63–0.77)
**180** **d**	0.703 (0.64–0.75)	0.711 (0.65–0.77)	0.723 (0.66–0.78)	0.706 (0.64–0.76)

Abbreviations: mDF, Maddrey discriminant function; NLR, neutrophil-to-leukocyte ratio; TSB, Trajectory of Serum Bilirubin.

## DISCUSSION

Most studies in AH have focused on sAH, which is associated with high mortality (2). However, there is limited available data regarding mAH concerning mortality and associated factors. In this global cohort study based on a registry involving 1845 patients with AH, we studied 446 patients suffering from AH. Compared to sAH, patients with mAH were older, a higher percentage were women, and a higher percentage were Caucasian or Hispanic/Latino patients. They also had fewer history of cirrhosis and were less likely to be admitted with renal failure. As expected, they also had lower severity scores and were less likely to receive corticosteroids than sAH. When evaluating the factors associated with increased mortality in patients with mAH, it is noteworthy that only age was associated with increased mortality.

Similar to findings in other studies, the mortality rates at 30, 90, and 180 days in patients with mAH are not low.^13^^,^^18^ In this study, the 30-day survival rate was 94.3%, but by 180 days, it dropped to only 87%. In this context, it is important to note that the perception of mAH as a mild disease is not accurate. Although the mortality rate in mAH is lower than in those with severe AH,^22^ it is higher than in decompensated patients with cirrhosis^23^ who do not develop AH and exceeds that observed in patients with other severe liver diseases, such as acute myocardial infarction (with a 30-day mortality of 6.2% in the United States)^24^ or community-acquired pneumonia (with a 30-d mortality of 4.0%).^25^^–^^27^ In our cohort, nearly 30% of patients did not have cirrhosis, suggesting that the increased mortality is largely driven by the acute inflammatory process characteristic of AH, even in its nonsevere form. In this sense, it seems important to consider that patients with mAH should have close outpatient follow-up, starting preventive measures to reduce the high mortality they reach up to 180 days. In addition, some previous studies have shown that patients with sAH (MELD score >20) have higher mortality compared to those with mAH. Additionally, in patients with sAH, variations in the MELD score are associated with increased mortality.^22^ In this study, we stratified patients with mAH according to different MELD score levels (8, 12, 15, 18, and 20) to determine if there is another level at which mortality differs, which could correspond to “mild” AH. However, the MELD score does not seem to have a direct effect on mortality. Variations in the MELD score seem to be less important in patients with mAH, and there does not appear to be a category of patients with “mild” AH.

Few studies have adequately evaluated the factors associated with mortality in mAH, which appears to have a different patient profile compared to sAH. Systematic reviews and studies evaluating the survival of these patients have often used data from clinical trials designed for sAH, which included some patients with mAH, and therefore have not been able to develop multivariable meta-regression models.^17^ One study, which included 590 hospitalized patients with mAH, evaluated only factors associated with 90-day mortality, finding that older age (HR: 1.03), corticosteroid use (HR: 1.8), and acute kidney injury (HR: 2.43) (95% CI: 1.33–4.47) were independently associated with 90-day mortality.^18^ In our study, the multivariate analysis for evaluating 30-day mortality showed that only age was associated with increased mortality in patients with mAH. This is interesting because, on one hand, patients with mAH are older compared to those with sAH, yet within this group, age is associated with higher mortality. This suggests that early management of infections may be crucial to prevent mortality in older patients with mAH. In our study, 30% of the patients used corticosteroids, but their use was not associated with a decrease in mortality. This finding is consistent with studies showing that corticosteroid use in patients with lower MELD scores does not have a clear benefit.^22^

Regarding the cause of death, it is notable that they follow the same distribution as previously published in patients with sAH,^22^ as well as the cause of death observed in the sAH group of this study. Multiorgan failure, infections, and esophageal varices bleeding are the main causes of death in this population. In this regard, and associated with the high mortality observed, early screening and treatment of infections in patients with mAH appear to be crucial. However, to date, studies evaluating the effect of prophylactic antibiotics in severe AH have not demonstrated a benefit in mortality.^10^ This, along with abstinence, seems to be an important measure to improve the survival of this group of patients.

Several scores have been published to assess the severity of AH.^8^ Traditionally, the mDF score was used to differentiate patients with higher mortality (sAH) from those with lower mortality.^14^ Currently, several studies have shown that MELD is the best score in sAH for classifying the severity of patients with AH, with the outcome being 30-day mortality.^15^^,^^28^ This score allows for the differentiation of patients who may benefit from the use of corticosteroids, with those having a MELD between 20 and 39 gaining the most benefit from this therapy.^22^ Recently, new scores have been evaluated, such as the TSB^19^ and NLR,^20^ which have performed well in determining the severity of sAH. However, evidence for the use of different scores in mAH is limited. Our study assessed the performance of different scores (MELD, mDF, TSB, NLR) none of which were ideal for stratifying patients with mAH, having AUCs between 0.671 and 0.733. Interestingly, the performance of the scores behaves differently between sAH and mAH, highlighting that the current way of evaluating patients with mAH is deficient. This study demonstrates that the new scores, TSB and NLR, can be effectively used in mAH, achieving similar performance to MELD and mDF. Notably, TSB showed better performance in predicting 30-day mortality. However, TSB is assessed over the first 7 days of admission and none of the scores have adequate performance in mAH. Therefore, new scores are needed to stratify patients with mAH and to better estimate their prognosis.^29^

Our study has several limitations. First, the retrospective design restricts our ability to comprehensively address potential confounding factors that may have influenced the outcomes. Second, the majority of patients were of Caucasian and Indian ethnicity, which could lead to the underrepresentation of other ethnic groups, thereby limiting the generalizability of our findings to certain minorities. In terms of causes of death, a significant proportion of patients died from multiorgan failure, which may obscure other underlying causes, such as infections or hepatic dysfunction, that can contribute to multiorgan failure. Another limitation is that some patients received corticosteroids based on the Maddrey score despite having a MELD score below 20. While this could affect the interpretation of mortality outcomes, this variable was included in the multivariable model. Additionally, we were unable to obtain records of alcohol consumption levels or determine whether patients achieved abstinence after episodes of AH. Furthermore, there was an underreporting of infections in patients with mAH, which prevented this variable from being included in the models. The use of MELD for patient selection may have also resulted in a reduced range of MELD scores, potentially leading to lower accuracy in predicting outcomes. Finally, the performance of other scoring systems based on similar parameters (bilirubin and coagulation factors) may also generate interactions and limit the interpretation of these results. Despite the limitations, the current study has multiple strengths. The current study included numerous patients from 15 centers across 8 countries and diverse ethnic backgrounds. We conducted long-term follow-up on patients. The evaluation of multiple scores and their performance in mAH is a novelty that could inspire new studies and guide clinicians’ decision-making.

In conclusion, patients with mAH tend to be older, more often women, of Caucasian and Hispanic/Latino ethnicity, and have a lower prevalence of cirrhosis. This condition is associated with high mortality rates at 30, 90, and 180 days, linked to multiorgan failure, infections, and esophageal varices bleeding, with factors associated with mortality being age and the presence of infections during hospitalization. The scores commonly used to stratify risk in sAH (MELD, mDF, and the new scores TSB and NLR) showed no differences among them; however, showed better prediction in stratifying mAH. It is crucial to recognize the severity of mAH to foster the development of new therapeutic options targeting this population.

## Supplementary Material
