# Supplementary material for: Moderate alcohol-associated hepatitis: A real-world multicenter study
Source: Hepatol Commun. 2025 Mar 24;9(4):e0673. doi: 10.1097/HC9.0000000000000673 (PMC11936654; doi:10.1097/HC9.0000000000000673)
Supplement: Supplementary file 1 [file hc9-9-e0673-s001.docx]

**SUPPLEMENTARY MATERIAL**

**Supplementary Table 1.- Centers and participants of the study**

| **Country** | **Center (n)** |
| --- | --- |
| **Colombia** | Hospital Pablo Tobón Uribe (HPTU) (20) |
|  | Hospital San Vicente Fundación Rionegro (6) |
| **Canada** | University of Alberta (29) |
| **Chile** | Pontificia Universidad Católica de Chile (26) |
|  | Hospital San Juan de Dios (7) |
|  | Hospital el Pino (33) |
| **Spain** | Hospital Gregorio Marañón (18) |
|  | Hospital Universitario Marqués de Valdecilla (69) |
|  | Hospital Universitari Vall d'Hebron (45) |
|  | Hospital de la Santa Creu i Sant Pau (65) |
| **India** | Institute of Liver and Biliary Sciences, New Delhi (442) |
| **Mexico** | Hospital General de México “Dr. Eduardo Liceaga” (40) |
| **Slovakia** | Faculty Hospital FD Roosevelt (21) |
| **United States** | Baylor College of Medicine, Houston (28) |
|  | University of South Dakota (138) |
|  | Mayo Clinic, Rochester (153) |
|  | Montefiore Medical Center, Bronx NY (10) |
|  | University of Florida (173) |
|  | University of Kansas Medical Center (499) |
|  | Yale University (23) |

**Supplementary Table 2.- Survival stratified by MELD**

| **Days of Follow-up** | **MELD** | **Survival** | **95% CI** |
| --- | --- | --- | --- |
| **30** | 8 | 94.4% | 82.4% - 98.8% |
|  | 12 | 95.7% | 92.8% - 98% |
|  | 15 | 95.1% | 92.7%-97.5% |
|  | 18 | 94.4% | 91.9%-96.8% |
|  | 20 | 93.9% | 91.1%-96.6% |
| **90** | 8 | 90.5% | 71.4% - 97.8% |
|  | 12 | 91.3% | 86.5%-95.2% |
|  | 15 | 90% | 86%-93.9% |
|  | 18 | 88.6% | 84.6%-92.6% |
|  | 20 | 87.6% | 83%-91.9% |
| **180** | 8 | 84.9% | 55% - 96.6% |
|  | 12 | 87.4% | 80.4%-93% |
|  | 15 | 85.6% | 80%-90.8% |
|  | 18 | 83.6% | 78.5%-88.7% |
|  | 20 | 82.2% | 76%-88.1% |
| CI: Confidence interval | | | |

**Suplementary Figure 1. Comparison of incidence curves - Mortality between a competing risk model with transplantation vs. a simple risk model.**

**
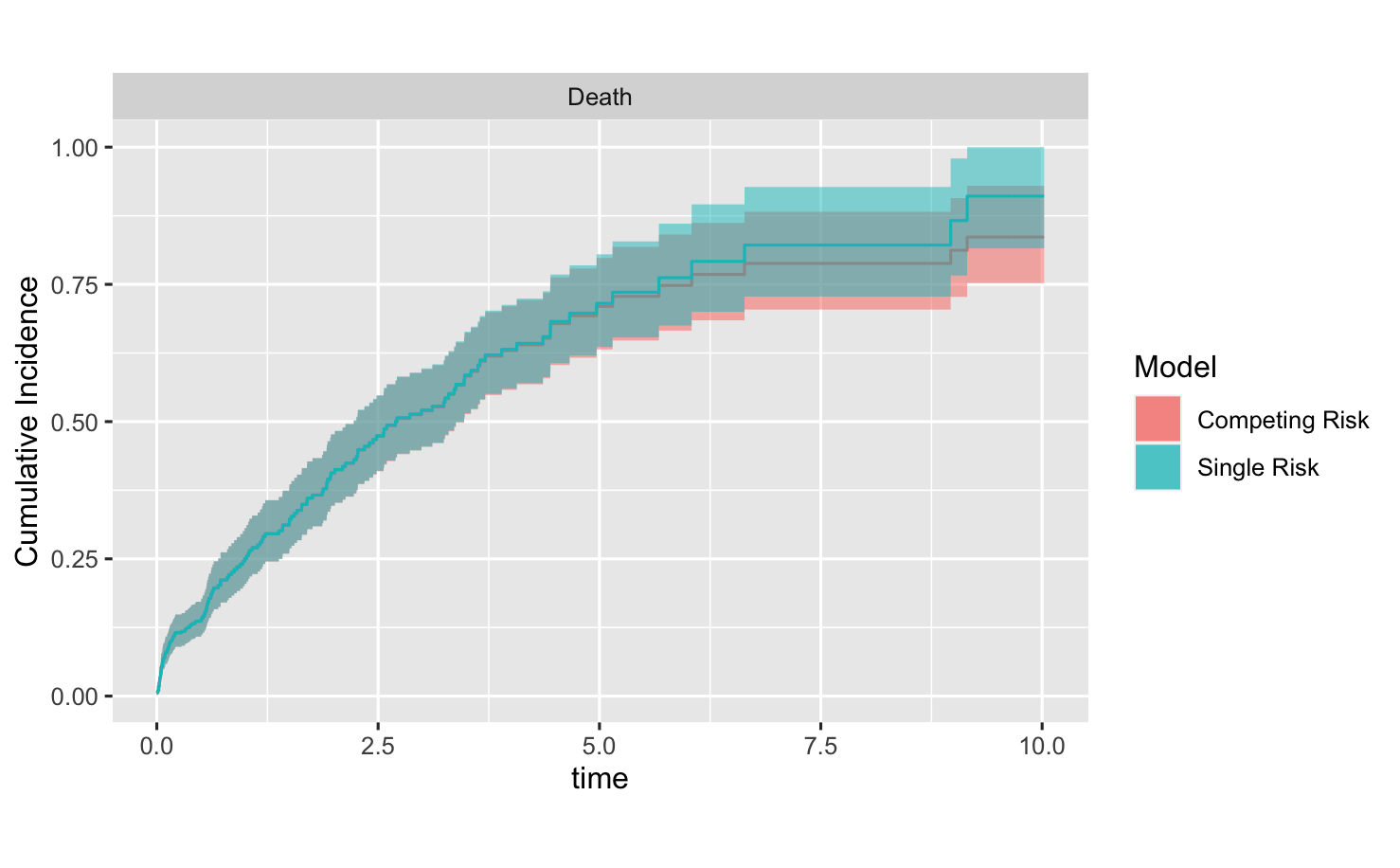
**

| **A** |
| --- |
| **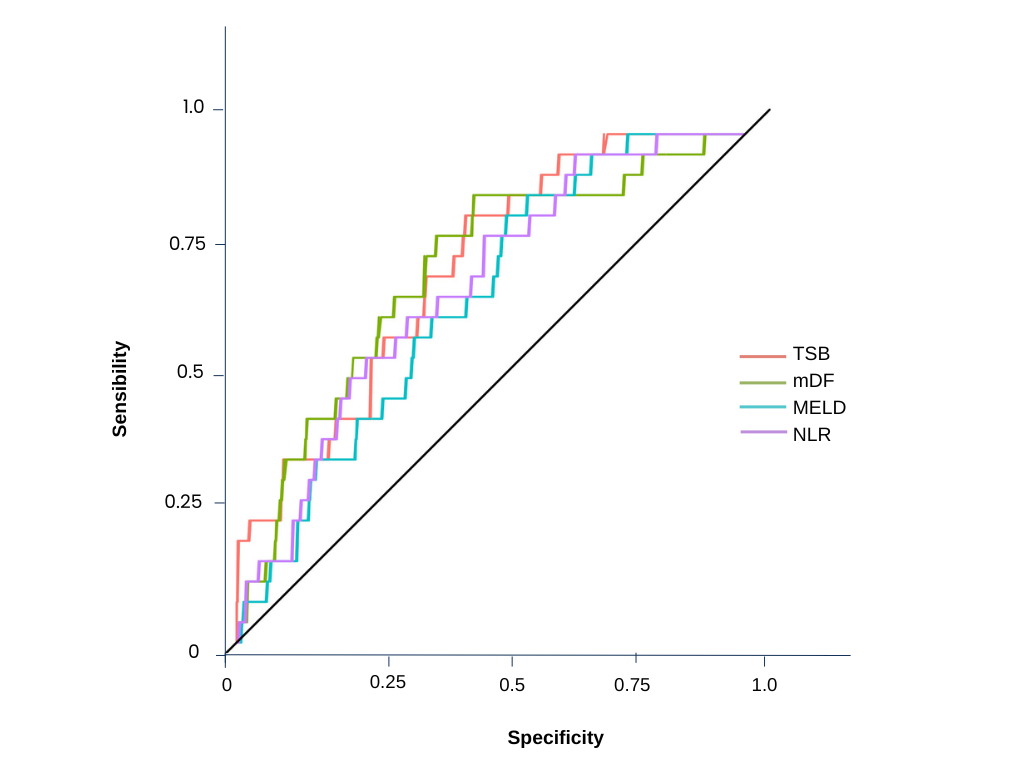** |
| **B** |
| **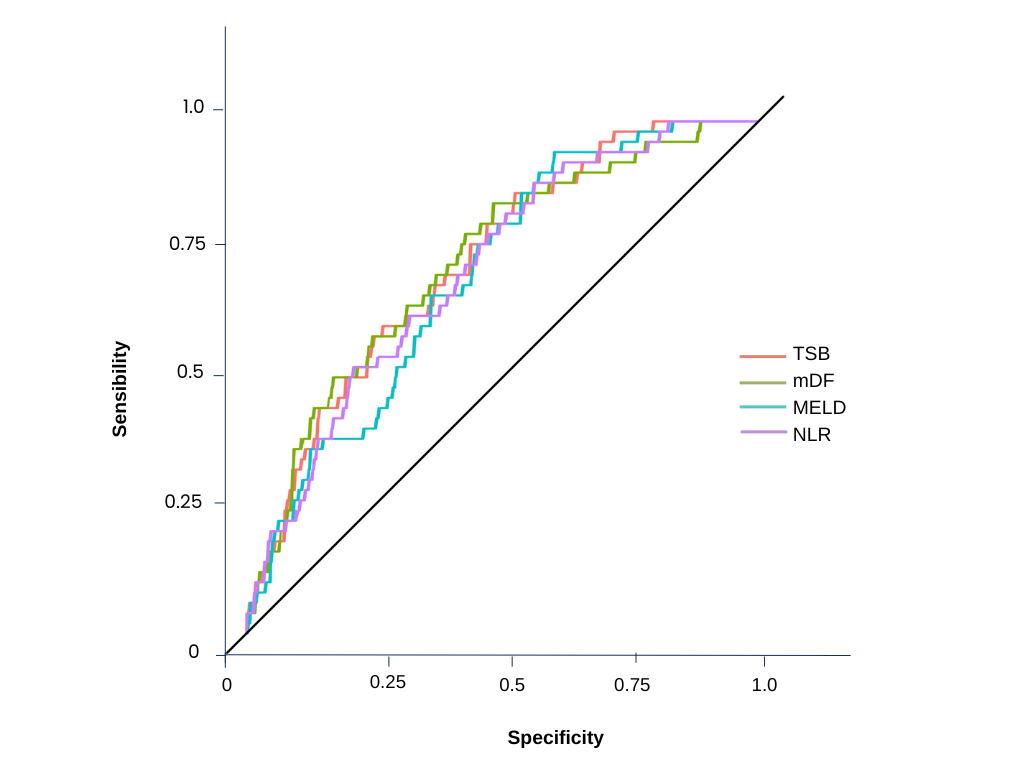** |
| **C** |
| **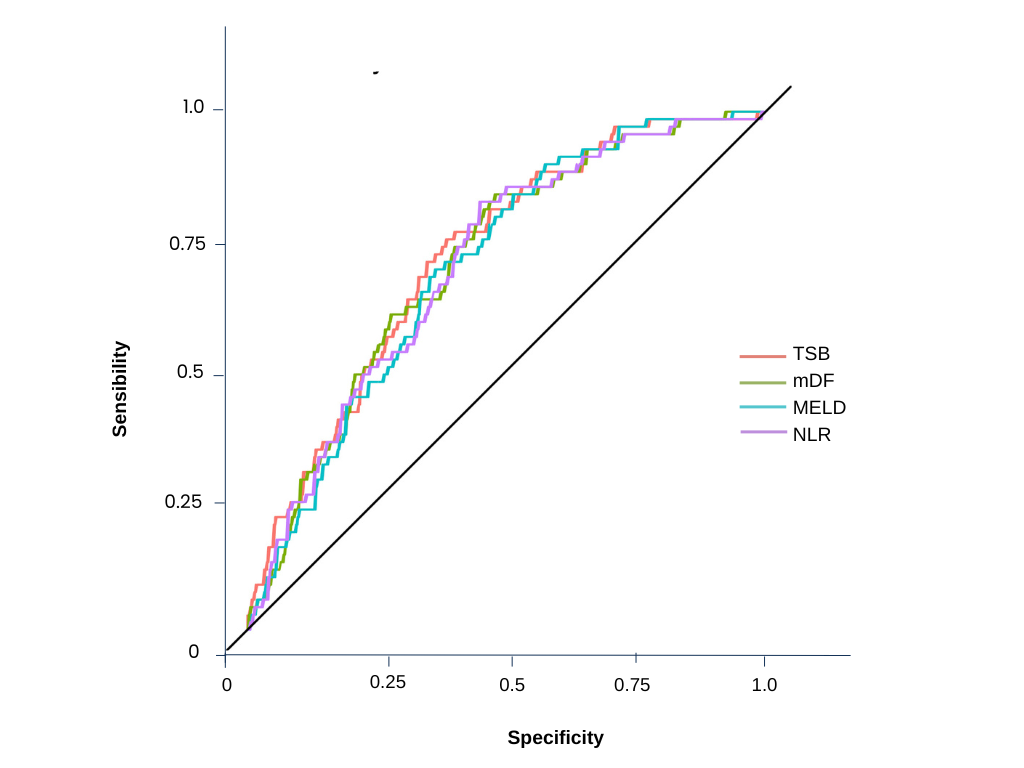** |

**Suplementary Figure 2. ROC curves to evaluate the performance of different scores in predicting mortality. A** 30-day mortality, **B** 90-day mortality, and **C** 180-day mortality. The scores assessed included TSB, mDF, MELD, and NLR. TSB: Trajectory of Serum Bilirubin; mDF: Maddrey's discriminant function; MELD: Model for End-Stage Liver Disease; NLR: neutrophil to leukocyte ratio.
